# Impact of the Provision of Safe Drinking Water on School Absence Rates in Cambodia: A Quasi-Experimental Study

**DOI:** 10.1371/journal.pone.0091847

**Published:** 2014-03-14

**Authors:** Paul R. Hunter, Helen Risebro, Marie Yen, Hélène Lefebvre, Chay Lo, Philippe Hartemann, Christophe Longuet, François Jaquenoud

**Affiliations:** 1 Norwich School of Medicine, University of East Anglia, Norwich, United Kingdom; 2 1001 fontaines pour demain, Caluire et Cuire, France; 3 Teuk Saat 1001, Phnom Penh, Cambodia; 4 Département Environnement et Santé Publique, Faculté de médecine de Nancy - Université de Lorraine, Nancy, France; 5 Fondation Mérieux, Lyon, France; CUNY, United States of America

## Abstract

**Background:**

Education is one of the most important drivers behind helping people in developing countries lift themselves out of poverty. However, even when schooling is available absenteeism rates can be high. Recently interest has focussed on whether or not WASH interventions can help reduce absenteeism in developing countries. However, none has focused exclusively on the role of drinking water provision. We report a study of the association between absenteeism and provision of treated water in containers into schools.

**Methods and Findings:**

We undertook a quasi-experimental longitudinal study of absenteeism rates in 8 schools, 4 of which received one 20 L container of treated drinking water per day. The water had been treated by filtration and ultraviolet disinfection. Weekly absenteeism rates were compared across all schools using negative binomial model in generalized estimating equations. There was a strong association with provision of free water and reduced absenteeism (Incidence rate ratio = 0.39 (95% Confidence Intervals 0.27–0.56)). However there was also a strong association with season (wet versus dry) and a significant interaction between receiving free water and season. In one of the intervention schools it was discovered that the water supplier was not fulfilling his contract and was not delivering sufficient water each week. In this school we showed a significant association between the number of water containers delivered each week and absenteeism (IRR = 0.98 95%CI 0.96–1.00).

**Conclusion:**

There appears to be a strong association between providing free safe drinking water and reduced absenteeism, though only in the dry season. The mechanism for this association is not clear but may in part be due to improved hydration leading to improved school experience for the children.

## Introduction

The receipt of a good quality education is one of the most important factors in enabling children to fulfil potential in later life and reduce poverty [1]. Increased educational attainment is also associated with substantial health gains especially on child health in future generations including reduction in child mortality [2], [3]. Important gains in child health may be associated even with future mothers improved access to primary education alone [3]. The importance of access to education is reflected within the Millennium Development Goals of the commitment to ensure that all children can complete a course of primary education [4]. In an earlier review of studies from developing countries, the author pointed out that time spent learning being linked to educational achievement is one of the most consistent findings [5]. However, as pointed out by Abadzi [1], instructional time available to children in many developing countries is often markedly reduced. Indeed Abadzi concluded that “assumptions about the pro-poor poverty alleviation effect of education may be unrealistic”, and that additional public investment may fail to mitigate poverty, unless it improves instructional delivery [1]. There are many reasons for this reduced educational contact time in low income countries, some of which are institutional such as teacher absenteeism, frequent school closures, etc [1]. However, even when schools are open, pupil absenteeism can be high [1]. Clearly reducing student absenteeism is important to improving educational attainment and consequent poverty alleviation.

Recent interest has turned towards the potential role of improving water and sanitation provision in schools as a tool towards improving children’s health and educational achievements. In a recent systematic review, the authors identified 41 studies that reported on the impact of water, hygiene and sanitation interventions on health and educational outcomes of which 8 were concerned with absenteeism [6]. Most of these studies were from developed nations. Although there was some indication of links between water and sanitation prevision and absenteeism, this was strongest around sanitation provision and absenteeism in menstruating girls. No strong conclusions could be made around the importance of drinking water. In probably the largest study of its type Freeman et al. reported on a large cluster randomised trial in Kenya on the impact of water, sanitation and hygiene interventions on absenteeism [7]. This study included over 6000 pupils from 135 schools in three study arms: a control with no intervention, a second study arm with hygiene promotion and chlorine for drinking water treatment and a third study arm with sanitation improvement in addition to the hygiene promotion and water treatment. The authors found no significant difference between any of the study arms, unless they did further sub-group analysis. Furthermore, by restricting their drinking water intervention to provision of near-to-use chlorination, Freeman et al. only tested the impact of drinking water disinfection and did not undertake an adequate assessment of the value of provision of drinking water per se in schools on educational outcomes [7]. Furthermore, serious doubts have been raised about the health value of household chlorination of drinking water as blinded studies have repeatedly found no benefit [8], [9]. The available evidence of the benefits or otherwise of drinking water interventions targeted at the level of the school in developing countries is, therefore, weak. We report a quasi-experimental study of the impact of provision of treated water in containers to schools on recorded absenteeism.

## Methods

This study was approved by the University of East Anglia Faculty of Health ethical committee and the Cambodian National Ethics Committee for Health Research. Given that we did not introduce any intervention, only summaries of routinely collected data were obtained and no person specific data was collected, there was no requirement for informed consent to be obtained.

The intervention being investigated was delivery of treated water in containers to schools. These water containers were provided free of charge to schools and funded by “1001 fontaines pour demain” (1001F), a non-governmental and not-for-profit organization based in Caluire, France. 1001F has been working in Cambodia since 2005. The basic model is to identify local entrepreneurs and financially support them to build a local plant to bottle filtered and ultraviolet disinfected water in cleaned and disinfected 20 L containers. Most of these containers are then sold to local customers. During and after start-up 1001F technical staff provide training and an ongoing quality assurance scheme. Funding for 1001F is mainly from private donors, though it has also received financial support from French Embassies in the countries where it works. A video highlighting 1001F’s work can be seen at the following link: http://fr.youtube.com/watch?v=8bykbVECVrE. In some of the villages, 1001F paid the entrepreneur to provide free water to the village school. Each participating school was provided with 1001F water in containers to be placed in the classroom so that each child could take water whenever they wished. For those schools participating in the scheme one 20 L bottle of water was delivered to each class each day. Given that the average class size was 38 children, this equates to approximately 0.53 L per child per day. The overall cost of the scheme was US$1.4 per child per year.

In this study, we obtained absenteeism data from the four schools where 1001F were providing free water. In addition we obtained this same data from four schools not in receipt of free water. In a related community study of childhood diarrhoea we were conducting a longitudinal study of childhood diarrhoea and water use in 25 villages. These villages had been chosen at random from all villages with an established 1001F presence or through a process of propensity score matching, the details of which is described elsewhere [10]. Four schools from these 25 villages were in receipt of the free school water scheme and willing and able to provide absenteeism data. Four control schools were chosen from the other 25 villages based on number of registered students present and the proportion of students under 14 years closest to those values of the intervention schools. The head teacher was then approached and invited to participate.

Data collection was based on routinely collected absenteeism data provided to the study team by the head teacher. Data was provided from the week beginning 4^th^ December 2011 to 31^st^ May 2012. This period spread over two school terms one of which was in the dry season and the other the wet season.

Data analysis was done using STATA version 11. Absenteeism rates per week were calculated as the number of days absent/(5×children registered). Random effects negative binomial regression analyses were done using a generalized linear model with a random intercept for school. The outcome variable was the number of days lost in each week from absenteeism and the number of children enrolled in the school was the exposure variable. The predictor variables were whether or not the school received water and season. Interaction terms were included for intervention and season.

In one school it was discovered that the number of water containers delivered fell short of the contracted amount. A further regression analysis was done for this school with days missed in the week being the dependent variable. The actual number of water containers delivered in the week and days missed at all the other schools combined were predictor variables. The analysis was restricted to the dry season and excluded holiday weeks.

## Results

Table 1 shows certain key characteristics of to the villages where each of the eight schools were based. It can be seen that across most characteristics the intervention and control schools were generally very similar. The main exception is that very few people in intervention villages have access to improved water or sanitation compared to the control villages. This is not too surprising as the 1001F had primarily targeted its intervention at schools in areas where it was known that the local community had poor access to improved drinking water. Also of note was that rather more of the populations of the intervention villages were reported as being migrants. The predominant source of drinking water in the control schools was whatever the children brought in from their home. In one control school (C2) children also had access to a hand pump and jar in a pagoda about 100 m from the school and in another (C3) there was a rainwater harvesting tank for which children were reported to have some use.

**Table 1:** Characteristics of the villages in which the schools were based.

School code	C1	C2	C3	C4	I1	I2	I3	I4
Number of children in school	379	438	267	450	587	271	954	174
Number of households in village	318	320	405	191	331	256	227	192
Population	1634	1706	1951	942	1378	1126	1075	873
% population male	49	50	48	51	51	50	49	49
% population <5 yrs old in village	8	10	11	13	8	12	12	10
% population 5 to 14 yrs old	20	27	25	26	19	28	25	24
% population with lower secondary education or greater	96	96	95	98	92	98	99	98
% adult female literacy rate	84	58	72	34	66	50	73	64
% working in primary sector (mainly agricultural)	68	96	81	87	85	94	66	94
% population migrants	23	4	32	20	57	48	36	65
% with access to improved water	48	72	1	63	5	0	2	2
% with access to sanitation	84	65	28	19	14	6	16	38

School codes prefixed with I received the free water and those prefixed by C did not. Data from The National Institute of Statistics, Ministry of Planning, Royal Government of Cambodia (http://www.nis.gov.kh/index.php/online-statistics/resultonline).

**Figure 1:**
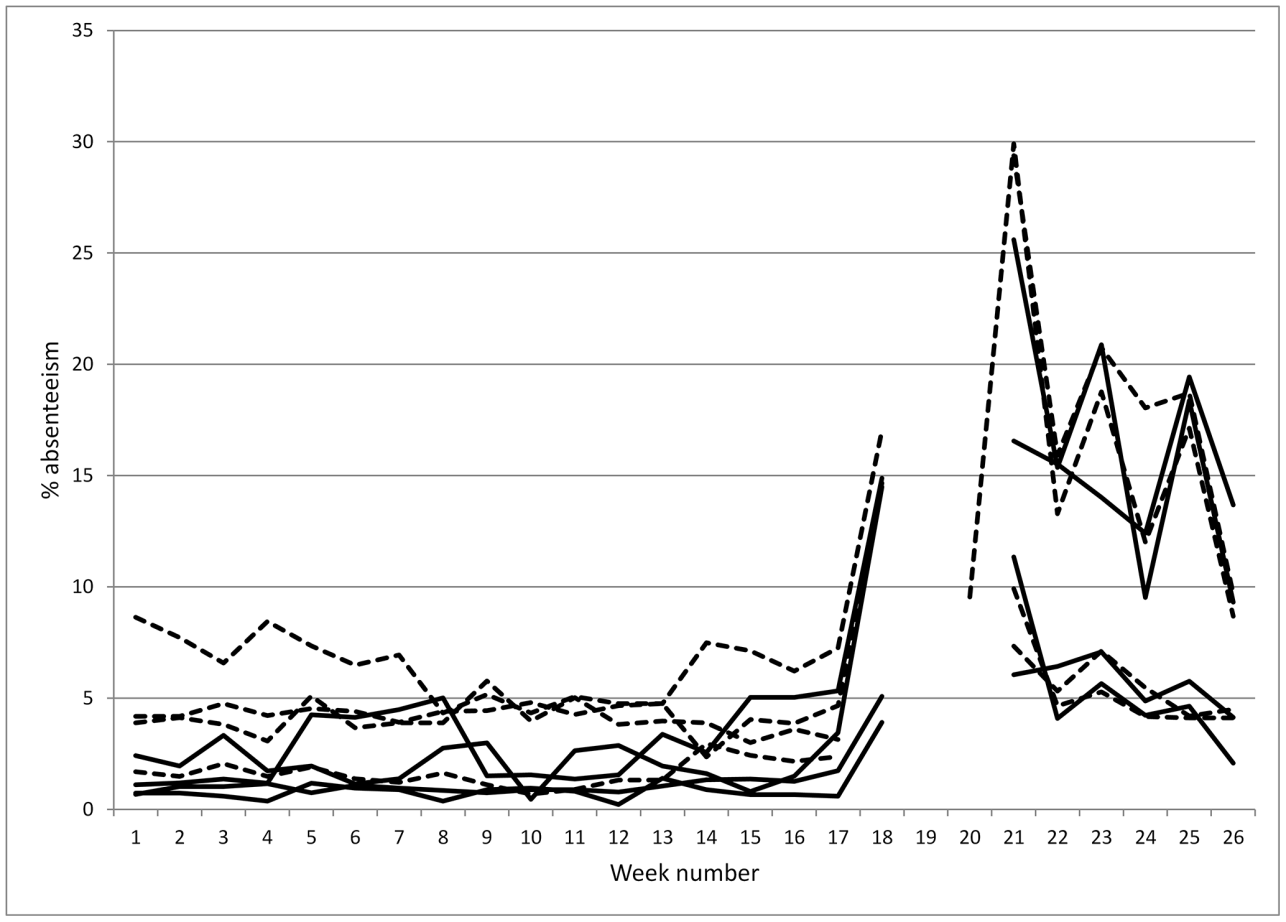
Absenteeism rate by school and week. Solid line shows rates for intervention schools and broken line for control schools.

Data was collected for 26 consecutive weeks. Three schools were closed during week 18, all schools were closed during week 19, and all but one in week 20. The dry season was taken to include all the weeks before the break in week 19 and the wet season in weeks subsequent to this holiday. Across all eight schools this represented 60,194 child weeks of follow-up. The overall absenteeism rate was 5.57%. Figure 1 shows the absenteeism rate for each school by week. The most obvious finding was the dramatic increase in absenteeism during the wet season, towards the end of the study period. This was not surprising given the fact that in many villages, children would be kept off school at this time to help in the fields.

Table 2 shows the results of the negative binomial regression analysis comparing absenteeism rates using whether or not the school received free 1001F water and season as predictor variables. In addition we investigated the interaction between season and receipt of 1001F water. It can be seen that absenteeism was less than half in the intervention schools compared to those who did not receive 1001F water. Given the significant interaction term the association between having 1001F water and reduced absenteeism was restricted to the dry season with no such association in the wet season (as was also clear in figure 1).

**Table 2:** Risk factors for absenteeism rates in schools.

Predictor		Incidence Rate Ratio	Lower 95% CI	Upper 95% CI	P
Receives 1001F Water	No	1			<0.001
	Yes	0.386	0.266	0.560	
Season	Dry	1			<0.001
	Wet	2.618	2.279	3.008	
Season-1001F water interaction	Yes-Wet	1.991	1.603	2.472	<0.001

At the end of the study period it became clear that one of the suppliers was not fully fulfilling their contract as they did not have sufficient capacity to provide water to the school and to their paying customers. Although container water was provided this fell short of the contracted amount. The remaining three schools received all their assigned supplies. Table 3 shows the results of the regression analysis of absenteeism in the school with incomplete water delivery adjusted for within week absenteeism in other participating schools. There was a significant association between the number of containers of water delivered in the week and reported absenteeism. For every extra container delivered there was a 2.9% reduction in absenteeism (95% confidence intervals (CI) 0.5 to 5.1%). The association was also tested between absenteeism and delivery in the previous week. Absenteeism was not associated with the number of containers delivered in the previous week.

**Table 3:** Risk factors for absenteeism in school with incomplete delivery of water containers during the dry season.

Predictor		Incidence Rate Ratio	Lower 95% CI	Upper 95% CI	P
Water delivered in week	/container	0.971	0.949	0.995	0.016
Absenteeism days in other schools	/days missed in week	1.000	0.992	1.007	0.976

## Discussion

In this study we have shown lower absenteeism in schools receiving free containers of 1001F water. However, this association was only seen in the dry season and not in the wet season. There were also strong seasonal effects as absenteeism in several of the schools increased dramatically during the wet season, irrespective of water delivery. We were informed that this increase in absenteeism during the early wet season was partly because children were frequently kept off school to help in the fields. We have, furthermore, shown that in one school where delivery of water containers fell short of the contracted amount, absenteeism rates were associated with the number of containers delivered in the week. As far as we are aware this is the first study to show that provision of adequate safe drinking water in school can affect attendance in a developing country.

Clearly one has to be cautious when interpreting the results of an observational study like this. Nevertheless, taking both analyses together, this gives a fairly strong indication that provision of safe palatable drinking water is indeed associated with reduced absenteeism. Firstly although this study was not blinded and so potentially open to some form of reporting bias, school absenteeism rates are not subjective and so our results should not be as at risk of reporting bias that has affected many other studies of water and health in low income countries [8]. We cannot, of course, exclude bias in the way the classroom teacher records the daily attendance register or in how the school compiles absenteeism data from the class registers. However, any such bias is far less likely when based on register records than may be expected by asking children to recall their absence history during interview as was done in the only other study of school absence and WASH [7]. Secondly, although it is plausible that selection of schools for the intervention may have led to a degree of bias, it is difficult to see how this would have affected the association found between number of containers delivered and absenteeism in the school with incomplete contract fulfilment. Of particular note here was that the intervention schools were generally in areas with poor domestic access to improved drinking water supplies and sanitation. If inadequate drinking water and sanitation does impact of school absenteeism, then if anything this source of bias would be expected to increase absenteeism in the intervention schools rather than reduce it. It is not clear what effect if any the greater number of migrants in some villages would have on absenteeism in school. We would however suggest that further randomised studies are required before a more definitive conclusion can be made.

This leaves the question of what was the mechanism between water supply provision and absenteeism. In this study we were not able to collect any data on the reasons for the absenteeism. Given the fact that the association was between absenteeism rates and water delivery in the same week and not the previous week, we are not suggesting that this association was primarily due to a reduction in waterborne infectious disease. A possible explanation in our view may be that by providing readily available palatable and safe water in the classroom, children are more likely to drink during the school day and so not become dehydrated. Even mild dehydration in vulnerable groups such as young children has been suggested as being associated with various adverse health effects [11]. Furthermore, in a recent study from a hot dry region of Italy, the authors showed that supplementary drinking water was associated with improved cognition and an improved subjective sense of vigour [12]. This Italian study is in line with similar findings from several previous researchers [13], [14]. What this suggests therefore is that provision of supplementary water sufficiently improves the child’s general wellbeing as well as the learning and experience of the school day as he/she is better hydrated. Consequently they are more likely to attend school the following day if they had felt good at school the previous day.

Even if, as we suspect, the main reason for the reduced absenteeism in the intervention group is due to improved hydration rather than a reduction in waterborne disease, this should not be taken as an indication that the provision of water of any quality would be acceptable. The link between contaminated drinking water and disease risk is well accepted and it is clear that the main risk falls on young children [15]. Any scheme to increase drinking water provision in the classroom that does not ensure that that water is safe to drink is likely to put the children at risk of waterborne disease. However, providing safe water in the school environment does not necessarily mean children will drink it. Indeed taste appears to be a major determinant affecting whether or not people continue to use safe drinking water sources [16], [17]. Chlorination of drinking water is associated with poorer taste for many people [18], [19]. On the other hand filtration can be associated with improved taste [20]. The fact that 1001F water uses filtration and Ultraviolet disinfection but not chlorination would mean that it would have better taste qualities than other safe water sources and so may be more likely to be used by children.

In conclusion, we have shown a significant association between provision of supplementary water in the classroom and reduced absenteeism rates. With the delivery mechanism in this study the cost per child is modest, but the potential benefit to children’s education and subsequent life potential could be extremely large. There is a great need for further research in this area, especially randomised control trials and studies aimed at determining the biological mechanisms behind this reduction in absenteeism.
